# Short De-Etiolation Increases the Rooting of VC801 Avocado Rootstock

**DOI:** 10.3390/plants9111481

**Published:** 2020-11-03

**Authors:** Zvi Duman, Gal Hadas-Brandwein, Avi Eliyahu, Eduard Belausov, Mohamad Abu-Abied, Yelena Yeselson, Adi Faigenboim, Amnon Lichter, Vered Irihimovitch, Einat Sadot

**Affiliations:** 1 The Institute of Plant Sciences, The Volcani Center, ARO, 68 HaMaccabim Road, Rishon LeZion 7528809, Israel; duman.zvi@gmail.com (Z.D.); gal.hadas@hadasbros.com (G.H.-B.); avi.eliyahu9@gmail.com (A.E.); eddy@volcani.agri.gov.il (E.B.); abuabied@agri.gov.il (M.A.-A.); elenae@volcani.agri.gov.il (Y.Y.); adif@volcani.agri.gov.il (A.F.); veredi@volcani.agri.gov.il (V.I.); 2 The Robert H. Smith Institute of Plant Sciences and Genetics in Agriculture, The Robert H. Smith Faculty of Agriculture, Food and Environment, The Hebrew University of Jerusalem, Rehovot 7610001, Israel; 3 The Institute of Post Harvest and Food Sciences, The Volcani Center, ARO, 68 HaMaccabim Road, Rishon LeZion 7528809, Israel; vtlicht@volcani.agri.gov.il

**Keywords:** avocado, adventitious root formation, etiolation, auxin, ethylene, cell wall

## Abstract

Dark-grown (etiolated) branches of many recalcitrant plant species root better than their green counterparts. Here it was hypothesized that changes in cell-wall properties and hormones occurring during etiolation contribute to rooting efficiency. Measurements of chlorophyll, carbohydrate and auxin contents, as well as tissue compression, histological analysis and gene-expression profiles were determined in etiolated and de-etiolated branches of the avocado rootstock VC801. Differences in chlorophyll content and tissue rigidity, and changes in xyloglucan and pectin in cambium and parenchyma cells were found. Interestingly, lignin and sugar contents were similar, suggesting that de-etiolated branches resemble the etiolated ones in this respect. Surprisingly, the branches that underwent short de-etiolation rooted better than the etiolated ones, and only a slight difference in IAA content between the two was observed. Gene-expression profiles revealed an increase in ethylene-responsive transcripts in the etiolated branches, which correlated with enrichment in xyloglucan hydrolases. In contrast, transcripts encoding pectin methylesterase and pectolyases were enriched in the de-etiolated branches. Taken together, it seems that the short de-etiolation period led to fine tuning of the conditions favoring adventitious root formation in terms of auxin–ethylene balance and cell-wall properties.

## 1. Introduction

The avocado (*Persea americana* Mill.) is an evergreen tree in the family Lauraceae, grown worldwide for its oil-rich, nutritious and popular fruit. The avocado species are traditionally divided into three botanical races: Mexican (*P. americana* var. *drymifolia* (Schlecht. & Cham.)), Guatemalan (*P. americana* var. *guatemalensis* L.Wms.), and West Indian (*P. americana* var. *americana* Mill.), which are originally from the Mexican and Guatemalan highlands, and the lowlands (coast) of Guatemala to Costa Rica, respectively [1]. Current commercial cultivars and rootstocks are based on selections among these three races and on hybrids within races. Archeological findings indicate that avocado has been domesticated since 6400 BC [1]. Although grafting has been practiced for over 2500 years [2], the use of grafted avocado trees has only been documented since 1911 after the discovery and introduction of the popular edible variety ‘Fuerte’ to California, USA [3]. Avocado is very sensitive to various types of soil stress, including salinity, lime, and nonaerated soil on the one hand, and dryness on the other, as well as to several soil pathogens, such as *Phytophthora cinnamomi* [3]. These sensitivities can be mitigated by grafting desired cultivars on selections of elite resistant rootstocks, a common concept used worldwide [4,5], which drove the wide-ranging rootstock survey carried out by Avraham Ben-Ya’acov in Israel, starting in 1962 [3,6,7,8]. This survey yielded an assortment of selected rootstocks which were labeled VC (vegetative clones), and were evaluated around the country with different cultivars as scions [8], and which are still in use [9,10]. The vegetative propagation of these elite rootstock clones requires induction of adventitious root formation from stem cuttings. However, since avocado is severely recalcitrant to rooting, induction of adventitious roots from avocado cuttings was a serious bottleneck in the industry of clonal rootstock propagation; it required the development of a complex method [11], which has been slightly modified [12,13,14] but is still the only one in use today. This method involves grafting of the desired rootstock (now as a scion) on a nurse seedling rootstock, and letting it grow in the absence of light to develop an etiolated branch. This branch is then partially covered with soil and induced to root by auxin and grafted again, now as the rootstock of a selected cultivar scion. The new seedling is then disconnected from the nurse rootstock and grown independently. According to this method, both the grafting on a juvenile seedling and etiolation are necessary to create the permissive conditions for adventitious root formation from an avocado branch, which is otherwise largely recalcitrant to the process. Other methods include only the etiolation step, in which etiolated branches are induced to root while still connected to the mother plant [14,15]. The essential physiological, molecular, and chemical contributions of the nurse seedling and the etiolation step to the process of induction and differentiation of the adventitious roots are still not entirely clear. It was recently found that an avocado scion grafted on a seedling rootstock exhibits lower expression of the microRNA miR172 than when grafted on a mature clonal rootstock [16]. Since miR172 is a maturation marker [17,18], this suggested transport of a juvenility signal from the seedling to the scion [16]. Indeed, transport of nucleic acids [19], hormones [20] and carbohydrates [21,22,23] through the graft zone has been documented and might participate in the support provided by the seedling rootstock to the etiolated branch’s ability to root. Etiolated branches of carnation [24] and pea [25] have been shown to contain higher levels of indole acetic acid (IAA), and in *Arabidopsis*, IAA levels increased 24 and 48 h after etiolated seedlings were transferred to light [26]. In all three cases, the etiolated branches exhibited improved rooting capabilities. On the other hand, a disadvantage was documented in etiolated *Arabidopsis* hypocotyls which contain decreased levels of soluble sugars [27], the latter playing a pivotal role in adventitious root formation [24,28,29].

The rooting abilities of branches from clonal avocado rootstock VC801, which was isolated in Israel and exhibits relatively high resistance to the soil pathogens *Phytophthora cinnamomi* and *Verticillium dahliae*, as well as to salty irrigation water [3,9,10] were investigated here. VC801 is a West Indian (*P. americana* var. *Americana*) variant which is highly recalcitrant to rooting. Data is presented here on the rooting abilities of etiolated and de-etiolated cuttings that have been disconnected from the branch grafted on the nurse seedling. Additionally, details are provided on the differences between etiolated and de-etiolated branches in terms of tissue organization, cell-wall composition, auxin and sugar accumulation, and gene-expression profiles to shed light on the mechanisms that might contribute to their rooting ability.

## 2. Results

### 2.1. Etiolated VC801 Avocado Cuttings Are Softer and Differ in Their Tissue and Cell-Wall Composition from De-Etiolated Branches

**Figure 1:**
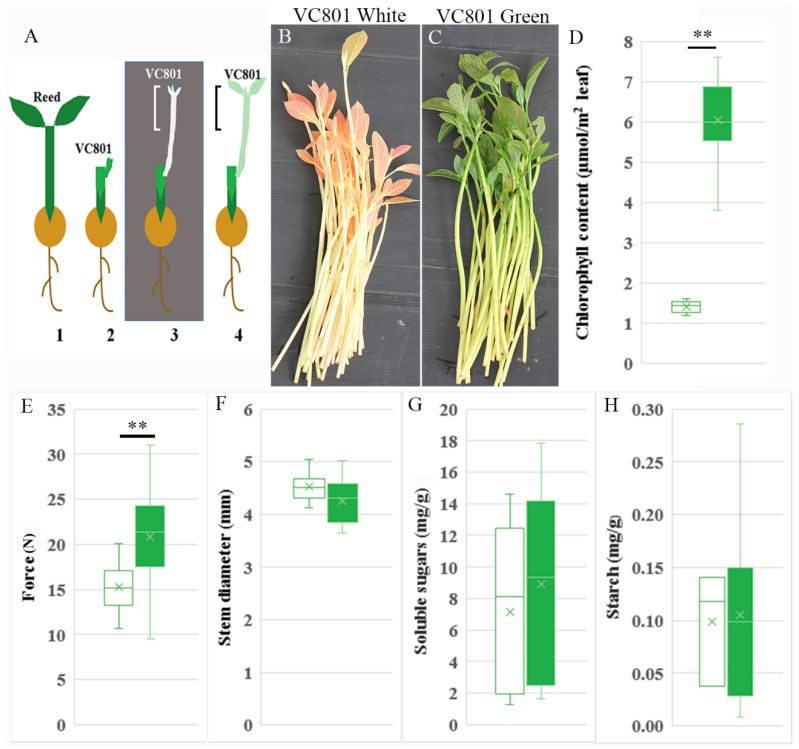
Avocado cuttings used in this study. (**A**) Schematic diagram of the grafted avocado from which the cuttings were taken. 1. A seedling of the nurse rootstock Reed. 2. Grafted VC801 branch with a bud on Reed. 3. The bud of VC801 is elongated in the dark to form an etiolated branch (white). 4. A seedling with an etiolated branch that was transferred to the light for 1–2 weeks (green). The marked portion in 3, and 4 is removed before the second grafting and shown in B and C, respectively. (**B**) Cuttings removed immediately after the etiolation step (1A3). (**C**) Cuttings removed after 1 week in the light (1A4). (**D**) Chlorophyll content. (**E**) Force required to compress the stems by 15% of their diameter. (**F**) Average diameters of the white and green stems. (**G**) Soluble sugar content (glucose, sucrose and fructose). (**H**) Starch content. Number of branches measured in **D**–**F** was 10–20, in **G**–**H** measurements were done in three replicates, in each three branches equal to 3 g were pooled. Asterisks indicate significant difference by Scheffe test at ** *p* < 0.01. White and green boxes refer to white and green branches, respectively.

This work was conducted in collaboration with the commercial nursery of Haskelberg in Kfar Vitkin, Israel. This is the biggest entity in Israel to perform the Frolich method [11] of double-grafting avocado plants for the creation of trees grown on vegetatively propagated elite selected rootstocks. The Haskelberg nursery grafts branches of the VC801 rootstock on various sources of nurse rootstock seedlings (for example, from the variety Reed), grows them in the dark, and after an etiolated branch is formed, its upper part—including the shoot apical meristem—is removed (Figure 1A) and the desired commercial varieties (Hass, Ettinger, etc.) are grafted on it. The remaining apical branches (Figure 1B,C) served as the biological system in the current research. The commercial interest in rooting the shoots removed before grafting lies in the ability to create two rooted vegetative rootstock branches out of the initial one grafted on the nurse seedling.

To compare etiolated to de-etiolated branches, parts of the plants were transferred to the light in a net-house for 1 or 2 weeks (green; Figure 1C) before the upper part of the shoot was removed. For uniformity during the experiments, chlorophyll was measured on the day branches were collected from the nursery: white (etiolated) branches had 1.39 ± 0.16 µmol chlorophyll/m^2^ leaf, and green (de-etiolated) branches had 6.05 ± 0.7 µmol/m^2^ (Figure 1D). To further characterize the differences between the white and green tissues, a compression test was performed to measure the maximal strain required to compress the stems by 15% of their diameter. The white branches were softer than the green ones (Figure 1E), while no significant difference was found between their widths, although the white ones were slightly wider (Figure 1F). Sugar content plays a role in adventitious root formation [24,28,29]. To check if, due to photosynthesis in the green branches, sugar content has changed, soluble sugars and starch were analyzed. It was found that white branches contained: sucrose 1.7 ± 0.22, glucose 6.6 ± 2.0, fructose 13.2 ± 1.2, and average of total soluble sugars 7.6 ± 1.8 mg/g; while green branches contained sucrose 2.3 ± 0.22, glucose 9.2 ± 0.74, fructose 15.1 ± 0.6, and average of total soluble sugars 8.9 ± 1.3 mg/g but, no significant differences were observed between the branches (Figure 1G). Of note, the starch content was much lower than that of soluble sugars (white branches 0.098 ± 0.03 mg/g, green branches 0.105 ± 0.04 mg/g) (Figure 1H), likely due to starch breakdown during dark [30].

**Figure 2:**
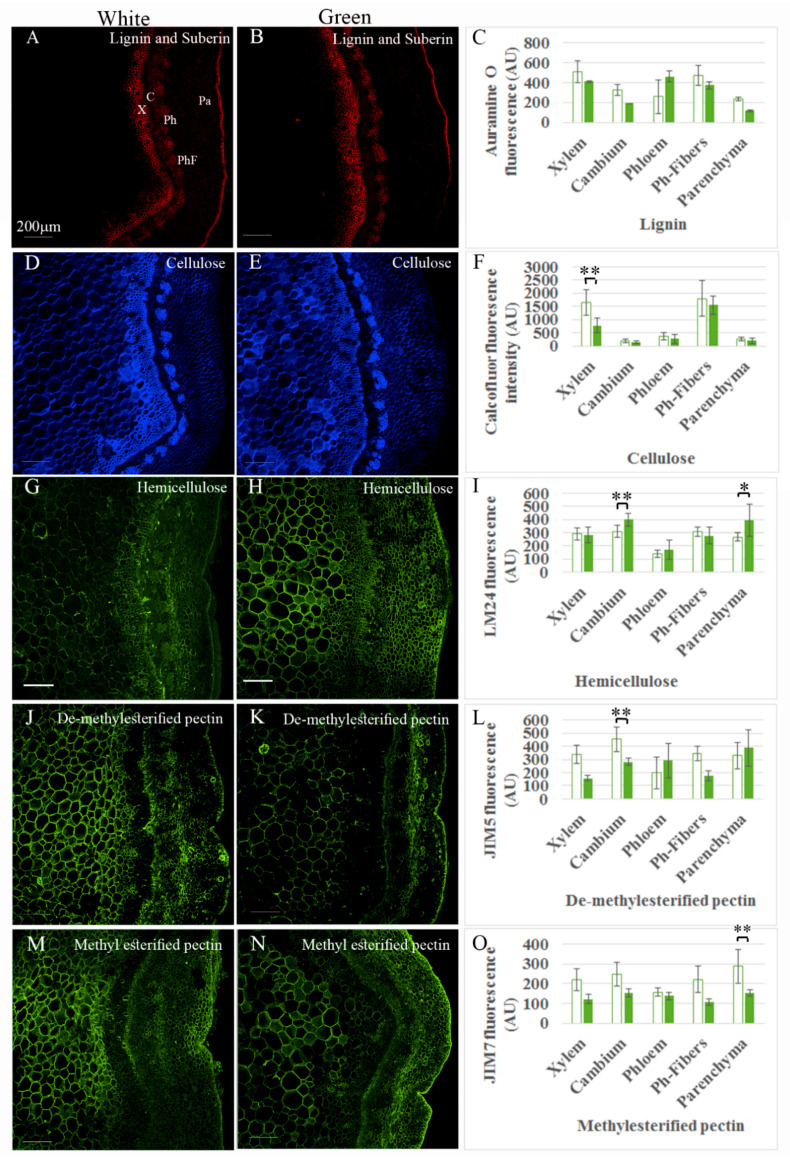
Fluorescence staining and measurements of fixed sections of the cutting bases. (**A**–**C**) Lignin and suberin staining by Auramine O. (**D**–**F**) Cellulose staining by calcofluor. (**G**–**I**) Hemicellulose staining with antibody LM24. (**J**–**L**) De-methylesterified pectin stained with antibody JIM5. (**M**–**O**) Methylesterified pectin stained with JIM7. * *p* < 0.05, ** *p* < 0.01 by Scheffe test. Scale bars are 200 μm. X—xylem, C—cambium, Ph—phloem, PhF—phloem fibers, Pa—parenchyma.

**Figure 3:**
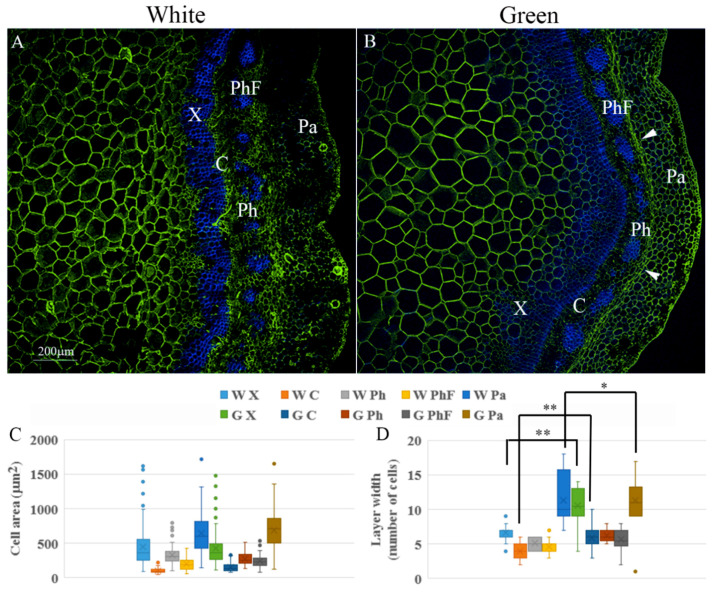
Changes in the different tissues in terms of cell size and number of cells along a line crossing each layer. (**A**) Representative section of a white branch stained with JIM5 (green) and calcofluor (blue). (**B**) Representative section of a green branch stained with LM24 (green) and calcofluor (blue). Arrowheads indicate collapsed cells. Scale bar is 200 μm. X—xylem, C—cambium, Ph—phloem, PhF—phloem fibers, Pa—parenchyma. (**C**) Chart showing average and distribution of cell areas. W—white branch, G—green branch. (**D**) Average number of cells along a line crossing each layer. Asterisks indicate significant difference by Scheffe test at * *p* < 0.05, ** *p* < 0.01.

Given that the branches were not dehydrated, the difference in firmness between white and green ones was most likely related to either the number of cells or the composition and thickness of the cell walls. To analyze the arrangement of the tissue, a histological analysis was performed. Sections were stained for the four major components of the cell wall: cellulose, lignin, hemicellulose, and pectin. Fluorescence intensity, as well as number of cells per column or number of cells along a line crossing the tissue, and cell area were measured in five different tissues: xylem, cambium, phloem, phloem fibers and cortical parenchyma. Staining was highly variable between branches, sections, and even in the same section, and fluorescence intensity varied among different regions of the same tissue (Figure 2). Therefore, differences observed in some sections were not confirmed as statistically significant when summarizing the data from many samples. Nevertheless, some conclusions could be drawn with good certainty. Despite previous reports on increased lignification in green tissues compared to etiolated ones [31,32], no significant change in Auramine O staining was observed after the short de-etiolation period, suggesting that the green branches preserved etiolation traits at this stage (Figure 2A–C). Calcofluor staining was significantly higher in xylem cells of the white vs. green branches (Figure 2D–F). Interestingly, the number of xylem cells increased significantly when the branches were transferred to the light (Figure 3A,B,D). This suggested an increase in xylem differentiation accompanied by elongation of tracheids and/or fibers with thinner cell walls [33,34] in the green branches. Accordingly, an increase in the number of cells in the cambium columns from 4 ± 1 to 6 ± 1.5 (Figure 3A,B,D and Figure S1) after transfer of the branches to the light could indicate on cambium activation [35], which might explain the addition of xylem cells. Fluorescence intensity of the anti-hemicellulose antibody was lower in the cambium and parenchyma of the white vs. green branches, suggesting either lower biosynthesis or higher activity of xyloglucan hydrolases during etiolation. Interestingly, the cell-wall composition of the cambium also differed in de-methylesterified pectin content according to JIM5 antibody staining, with higher intensity in the white branches (Figure 2G–L). Parenchymal cells of the white branches showed higher fluorescence intensity derived from JIM7 antibody, suggesting higher content of methylesterified pectin (Figure 2M–O).

The increase in the number of cells in the xylem tissue seemed to press the outer layers outward, leading to the crushing and collapse [33] of a cell layer in the older region of the phloem or nearby parenchymal cells in many of the green branches (Figure 3B, arrows). This might have contributed to the narrowing of the cortical parenchymal region in some green branches (Figure 3B,D). No significant differences were measured in the average cell areas of the different tissues, excluding the crushed cells (Figure 3A–C).

Taken together, the exposure of the etiolated branches to light led, in addition to an increase in chlorophyll contents, to changes in cellular organization and cell-wall composition (for example, an increase in the number of xylem cells and in hemicellulose content). These changes might contribute to the increased rigidity of the green branches (Figure 1E).

### 2.2. Induction of Adventitious Root Formation in Etiolated and De-Etiolated Branches of VC801

**Figure 4:**
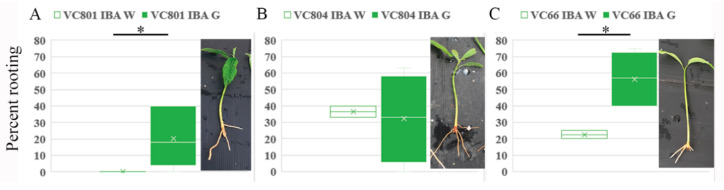
Induction of adventitious root formation in white and green branches. Branches (about 20 per treatment) were incubated in 60 ppm K-IBA by submerging the cutting base for 24 h. Rooting rates of VC801 (**A**), VC804 (**B**), and VC66 (**C**) were measured after 1 to 2 months. Asterisks indicate significant difference by Scheffe test at * *p* < 0.05.

High variability in rooting capability between clones of the same species is a well-known phenomenon [15]. Among the clonal VC rootstock collection, VC801 is considered to be more difficult to root than others. White and green cuttings of VC801 were incubated with 60 mg/L K-IBA for 24 h and then transferred to rooting tables for 1–2 months. The best rooting rates (5–40%) were obtained with the green branches, while the white branches did not root at all (Figure 4). Following similar rooting treatments, better rooting capability of green vs. white branches has been obtained for other vegetative rootstocks (both West Indian in origin): VC804, which was the second-best tolerant rootstock in the *Verticillium dahliae* and salinity survey mentioned above [9,10] exhibited 33–40% and 5–58% rooting for white vs. green branches, respectively, and VC66, which is considered to be easier to root and exhibited 20–25% and 40–75% rooting for white vs. green branches, respectively. To shed more light on the accumulation of auxin in VC801 etiolated and de-etiolated branches endogenous auxin and related gene-expression profiles were determined.

### 2.3. Hormone and Transcript Profiles of VC801 Etiolated and De-Etiolated Branches

**Figure 5:**
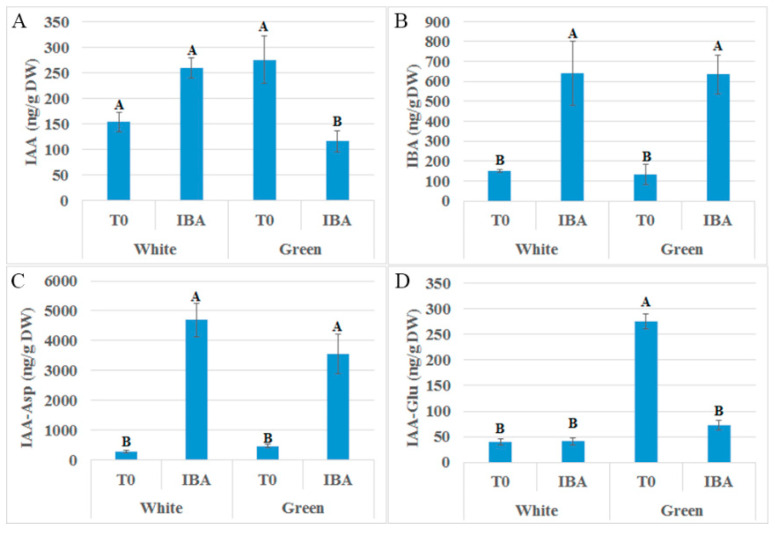
Auxin analysis. Cuttings were treated for 24 h with 60 ppm K-IBA and then incubated on rooting tables for an additional 24 h. Samples were quick-frozen in liquid nitrogen, ground and lyophilized at −50 °C. Auxin content was determined in three replicates. (**A**) IAA, (**B**) IBA, (**C**) IAA-Asp, (**D**) IAA-Glu. T0, before K-IBA treatment; IBA, treated cuttings. Bars with different letters show significantly different results by Scheffe analysis *p* < 0.05.

Etiolated (white) or de-etiolated (green) branches of VC801 were treated with 60 mg/L K-IBA for 24 h, transferred to rooting tables for another 24 h, and then quick-frozen in liquid nitrogen. Samples were analyzed for auxin concentrations, or were subjected to RNA isolation and sequencing. Endogenous IAA levels were found to be slightly higher in the green branches, although the difference was not statistically significant (Figure 5A). However, after IBA treatment, IAA levels were lower in the green branches than in the white ones. No differences in IBA or IAA–Asp were found between the white and green branches (Figure 5B,C). However, significantly higher IAA–Glu levels accumulated in the green branches before IBA application, which might be related to the higher IAA levels found in these branches (Figure 5D). Conjugates of IAA to Asp and Glu are thought to be directed to degradation [36], suggesting the balancing of excess auxin levels in the green branches. Of note, the wounding itself can lead to changes in auxin accumulation, and be different between etiolated and de-etiolated branches; however, this was not addressed in the current study.

**Figure 6:**
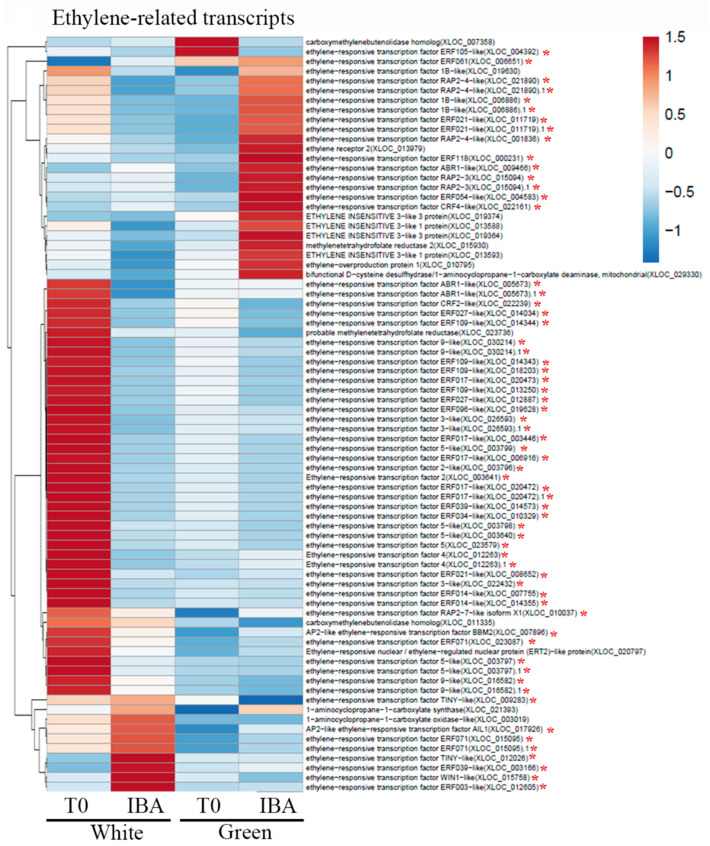
Heatmap analysis. Differentially expressed genes were scored for transcripts related to ethylene biosynthesis. Red asterisks show ethylene responsive transcription factors.

**Figure 7:**
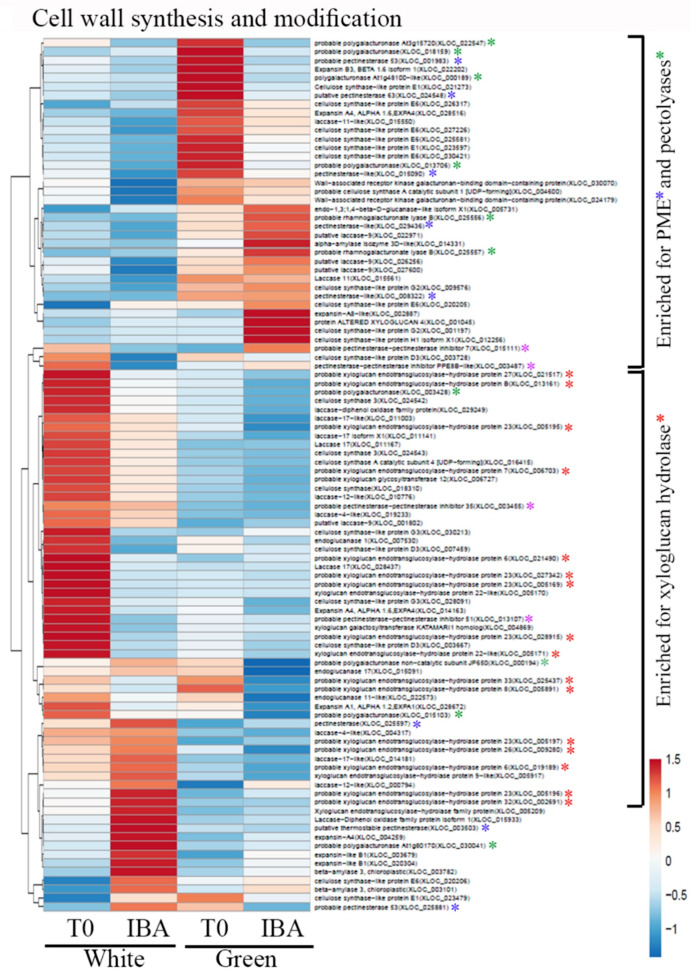
Heatmap analysis. Differentially expressed genes were scored for transcripts related to cell wall-modifying enzymes. Red asterisks show xyloglucan endotransglucosylases/hydrolases, blue asterisks—pectin methylesterase (PME), green asterisks—pectolyases such as polygalacturonase and rhamnogalaturonases, and purple asterisks—pectin methylesterase inhibitor PMEI.

For expression profiling, RNA was extracted from three replicates of either white or green pooled branches before treatment and 24 h after IBA treatment. Reads (between 15,986,718 and 25,326,487 of the different samples) exhibited 78.1–80.8% mapping (Figure S2A) to the recently published genome of the hybrid cultivar Hass (Mexican with 39% introgression of Guatemalan) [37]. The clustering of the four groups of treatments is shown in Figure S2B. Comparison of the transcriptomes revealed 2371 and 1967 elements included exclusively in nontreated (T0) and IBA-treated branches, respectively. In addition, 3806 and 3351 elements differed between white and green branches at T0 and after IBA treatment, respectively (at least 2-fold change, adjusted *p* < 0.05). Heatmap analysis of transcripts related to auxin metabolism, transport and signaling revealed differential patterns of expression between white or green branches, at T0 vs. 24 h after wounding and IBA treatment. The vast majority of differentially expressed transcripts were related to auxin signaling (Figure S3). These changes in auxin-related transcripts might underlie the differences in rooting capability, but the specific transcripts still need to be determined. Since darkness induces ethylene production [38], which has an influence on adventitious root formation [39,40], an additional heatmap analyzing differentially expressed ethylene-related transcripts was constructed. Figure 6 shows multiple ethylene-responsive transcription factors that were highly expressed in the white branches at T0. In contrast, the green branches at T0 expressed these transcripts at much lower levels. After wounding and IBA treatment, fewer ethylene-responsive transcription factors were induced in the green and white branches. Taken together, the green branches at T0 seemed to express the lowest number of ethylene-responsive genes. Ethylene is known to be involved in cell-wall modifications during fruit ripening [41] and formation of an abscission zone [42], both of which lead to cell-wall loosening. Heatmap analysis of cell wall-related transcripts indeed showed enrichment of xyloglucan endotransglucosylase/hydrolase expression in the white branches at T0 (Figure 7). The green branches, on the other hand, were enriched with transcripts related to pectin-modifying enzymes: pectin methylesterase, and pectolyases such as polygalacturonase and rhamnogalacturonate lyase (Figure 7). This might be related to the decrease in hemicellulose contents seen in the cambium and cortical parenchyma of the white branches (Figure 2I), and the decrease in methy-lesterified pectin in the cortical parenchyma of the green branches (Figure 2O). In addition, since de-methyl-esterified pectin is prone to cleavage by pectolyases [43], this might underlie the reduction in JIM5 staining (de-methylesterified pectin) in the cambium of green branches. Taken together, differences in ethylene production in the white branches might lead to changes in cell-wall properties, which in turn might contribute to the rooting capabilities of these branches [44].

## 3. Discussion

Here, it is shown that short de-etiolation provides an advantage for adventitious root formation in shoots of the clonal avocado rootstock VC801 (as well as VC804 and VC66) which were removed from the nurse seedlings on which they were grafted. It is proposed that the short de-etiolation leads to changes in chlorophyll contents, cell-wall composition, and hormone profiles that compensate for the loss of support from the nurse seedling; at the same time, the advantages provided by etiolation are not completely eliminated. The results presented here suggest that the sum of changes leads to activation of the cambium, as detected here by the increase in cambial cells per column, and fine-tuned optimization of the conditions for adventitious root formation in these branches. Interestingly, the increase in cambial cells seemed to be accompanied by an increase in xylem cells in the green branches. It has been previously argued that activation of the cambium, which promotes xylem differentiation, counteracts the differentiation of adventitious roots in the *rac* mutant of *Nicotiana tabacum* [45]. In the current work, the formation of adventitious roots increased.

Several potential causes are suggested here which might be involved in the improved rooting abilities of the short-term de-etiolated branches.

The first is auxin content and perception. Slightly higher concentrations of IAA accumulated in the de-etiolated green shoots of VC801, which also showed better rooting ability, compared to the white etiolated shoots. This is in agreement with a previous finding in *Arabidopsis*, where IAA levels increased upon exposure of etiolated seedlings to light [26]. Auxin is a major player in adventitious root formation and IBA is the main rooting enhancer used today [39,40]. IBA is a precursor of IAA [46,47], and accumulation of the latter in the cutting base has been shown to be critical for adventitious root formation [28]. Analysis of endogenous IAA in various plants has revealed higher concentrations in correlation with better rooting; for example, in the easily rooted juvenile cuttings of *Eucalyptus grandis* compared to the difficult-to-root mature ones [48], and in young *Pisum sativum* cuttings compared to old ones [49]. The rooting rate of *Arabidopsis* leaf explants is slower under light vs. dark conditions, in correlation with lower IAA levels accumulated under the former conditions [50]. On the other hand, higher levels of IAA were detected in mature difficult-to-root shoots than in juvenile easily rooted shoots of chestnut [51], suggesting that IAA level is not always a limiting factor. In addition, using ^14^C-labeled IAA or NAA, no differences in auxin uptake or transport were found between rooting-competent and incompetent cuttings from loblloly pine [52], suggesting that most differences lie in auxin perception and signaling, and not in accumulated levels. In agreement with this insight, most of the differentially expressed transcripts between etiolated and de-etiolated branches that were related to auxin were auxin-signaling elements and not transcripts related to metabolism or transport. Nevertheless, problems in local coherent polar auxin transport have been proposed in difficult-to-root pine cuttings [53] and *Eucalyptus brachyphylla* [54]. In the latter it was shown that instead of roots, callus was formed which contained sporadic tracheary elements in random orientations [54]. It was concluded that auxin polar transport and canalization which are important for coherent xylem differentiation and restoration of proper vasculature [55,56,57] might be impaired in difficult-to-root plants.

In *Arabidopsis*, root organogenesis and callus formation share a similar mechanism at initiation, involving *LATERAL ORGAN BOUNDARIES DOMAIN* (*LBD* or *LOB*) transcription factors [58], the avocado counterparts of which are differentially expressed between white and green branches. It has also been shown that the switch between callus and root induction has to do with auxin levels; while high auxin promotes callus, lower levels promote root formation [58]. In avocado, roots were formed directly from the stems. However, the more stems with roots but no callus were obtained, the more stems exhibiting callus with no roots were also observed compared to non-responsive stems in the same treated group. In this respect, it is interesting to note that callus/adventitious root formation relationships are complex. In olive, for example, it was shown that callus formation preceded adventitious root formation; however, it was insensitive to rooting inhibitor [59,60,61]. Taken together, problems in auxin perception and polar auxin transport may exist in VC801 branches, leading to low rate of rooting or callus formation.

The second cause might involve ethylene, a stress-related phytohormone that is induced by wounding, darkness, water-logging and more [62]. The increased expression of ethylene-related transcripts in the etiolated branches described here suggests higher ethylene activity in those branches.

Of note, the transcriptomic data presented here are based on pooled samples. This approach was taken in order to reduce variability between the samples collected from a commercial nursery. A similar approach was taken in another research dealing with trees [63], and was recently validated to be useful [64].

A well-known effect of ethylene on plant growth has been dubbed the “triple response”: inhibition of hypocotyl and root-cell elongation, radial swelling of the hypocotyl, and enhanced curvature of the apical hook in seedlings [65]. The slightly larger diameter of the etiolated branches might be related to ethylene activity. In addition, the release of cell-elongation inhibition when the plants are transferred to the light might underlie the elongation of tracheids or xylem fibers, leading to the appearance of cells with thinner cell walls, as observed by calcofluor staining. Ethylene has been shown to induce adventitious root formation in rice [66] and tomato [67,68]. Although the precise molecular mechanism is not clear [69], ethylene’s ability to induce auxin biosynthesis [70,71] might be part of it. Interestingly, ethylene is involved in cell-wall remodeling in the abscission zone [42], and abscission components have been found to participate in cell separation for lateral root emergence by regulating the expression of cell wall-modifying enzymes such as polygalacturonases, which act on pectin [72]. In addition, the expression of xyloglucan endotransglucosylases/hydrolases and α-expansins is increased by ethylene in mung bean roots [73], both of which increase cell-wall extensibility. Furthermore, xyloglucan endotransglucosylases/hydrolases are highly expressed in *Arabidopsis* shoot apical meristem [74]. The *xx1/xx2* mutant, hampered in xyloglucan synthesis [75], and the *xyl1* mutant which lacks β-D-xylosidases [76] both have a distinct phyllotaxis phenotype [77], suggesting that xyloglucan homeostasis is important for proper meristem activity. In agreement with this, *xx1/xx2* forms more adventitious roots than wild-type plants [44]. Given that cell walls’ mechanical properties play a role in lateral organ formation [44,78], the finding of softer white branches with enriched expression of xyloglucan endotransglucosylases/hydrolases compared to green branches might be relevant for adventitious root formation in avocado. The green branches, on the other hand, exhibited relative high expression of pectin methylesterases (PME) and polygalacturonases, suggesting demethylation of pectin, which makes it more prone to degradation by polygalacturonases [43]. Local PME activity was previously shown to promote the formation of a lateral organ from the shoot apical meristem [79]. In this respect, it is interesting to note that the mutant *atpme3-1*, exhibiting reduced activity of pectin methylesterase (PME) and changes in methylesterification of galacturonic acid, exhibits increased formation of adventitious roots [80]. The above changes in expression profile were nicely correlated with a reduction in xyloglucans stained with LM24 antibody in the cambium and parenchyma of white branches, a decrease in de-methylesterified pectin (JIM5) in the cambium of green branches, and a reduction in methylesterified pectin (JIM7) in green branch parenchyma.

## 4. Materials and Methods

### 4.1. Plant Material

Shoots of avocado (*Persea americana* Mill.) rootstocks were provided by the Haskelberg nurseries (Kfar Vitkin, Israel). The decision to focus on the VC801 rootstock [3] was based on a recent rootstock survey performed in Gilat, Israel, the southern research station of the Agricultural Research Organization, in which VC801 exhibited relatively high resistance to *Verticillium dahliae* as well as to salty irrigation water [9,10]. The shoots of the VC rootstocks were grafted on seedlings of the Reed rootstock [3] and grown in the dark for 4–8 weeks until they reached a length of 20–35 cm (etiolated-white branches). Part of the plants were transferred to the light (nursery net house) for 1–2 weeks before the upper part of the shoots (~15 cm) was removed, (de-etiolated-green branches). The green and white branches were collected to the lab on the same day for further studies. Other rootstocks examined only for adventitious root formation under similar conditions were, VC804 and VC66.

### 4.2. Fluorescent Staining

For cellulose or lignin staining, transverse sections (0.3–0.5 mm) were obtained manually using a razor blade, fixed for 1 h in FAA (formaldehyde:ethanol:acetic acid, 10:50:5% v/v) supplemented with 150 µg/mL ascorbic acid, 1 mg/mL dithiothreitol and 10 mg/mL polyvinylpyrrolidone to prevent tissue oxidation. The samples were washed three times in phosphate buffered saline (PBS) and cleared using ClearSee as previously described [81]. Briefly, samples were incubated in ClearSee (10% *w/v* xylitol, 15% *w/v* sodium deoxycholate, 25% *w/v* urea in H_2_O) at room temperature under gentle agitation overnight. The samples were stained for cell walls as previously described [82]: samples were incubated for 5 min in 0.5% (w/v) Auramine O/ClearSee, rinsed twice with ClearSee and then placed on a slide with 1% (v/v) 1 g/L calcofluor white and 0.5 g/L Evans blue (Fluka Analytical 18909), and inspected by confocal microscopy. For pectin and hemicellulose staining, specific antibodies were used. Manually sectioned samples were fixed in freshly prepared 8% paraformaldehyde in buffer containing 100 mM PIPES pH 6.89, 5 mM MgSO_4_, 0.5 mM EGTA, and 1% Triton X-100. The samples were then rinsed three times (15 min each) in the same buffer without the fixative. For blocking, the samples were incubated in PBS containing 3% (w/v) bovine serum albumin (BSA, Sigma) for 1 h. This was followed by incubation with the following antibodies: JIM5 and LM19 for de-methylesterified pectin, JIM7 and LM20 for methylesterified pectin, and LM15, LM24, and LM25 for different epitopes of xyloglucan. JIM5, JIM7 and LM24 were found to work best for staining avocado tissues. Antibodies were diluted 1:10 in PBS with 3% BSA and incubated with the sections overnight in a humidified chamber. The next day, samples were washed three times with PBS, 15 min each, and incubated for 1 h with the secondary goat anti-rat antibodies conjugated to Alexa fluor 488 (Jackson ImmunoResearch Laboratories, USA). Then three washes with PBS were performed, with 1% calcofluor solution added to the third wash as an internal standard. An additional wash with distilled water preceded mounting in Fluoro-Gel mounting medium with DABCO antifade solution (Electron Microscopy Sciences, USA). The histological analysis was performed three times, in each 4–5 white stems, as well as 4–5 green stems were used, from each 10–20 manual sections were made.

### 4.3. Confocal Microscopy and Image Analysis

Samples were imaged in a Leica SP8 confocal laser-scanning microscope. Calcofluor and Auramine O were excited with 405 nm laser. Calcofluor emission was captured between 430–460 nm, and that of Auramine O, above 520 nm. The antibody-stained samples were excited at 488 nm and emission acquisition was at 505–535 nm. For fluorescence measurements, all image-acquisition parameters were equal, including the depth of the optical section. Fluorescence intensity in equal regions of interest, as well as cell sizes, were measured using Image J software.

### 4.4. Induction of Adventitious Roots

Shoots were excised, placed in a humidified cooler box and brought to a climate-controlled greenhouse within 2 h. Each cutting was 10–15 cm long, and two-thirds of each blade was excised to minimize evapotranspiration. The cutting bases were submerged for 24 h in 60 mg/L indole-3-butyric acid potassium salt (K-IBA, Sigma). The cuttings were planted in rooting medium containing peat, vermiculite and polystyrene flakes at a ratio of 1:2:3, respectively, on a heated (25 °C) rooting table under 90% humidity. Fungicides were applied to the rooting media on a weekly basis. Rooting percentage, was measured after 1 or 2 months.

### 4.5. RNA Isolation, RNA Sequencing and Bioinformatics

At least 10 different branches (including stem and leaves) were poled and quickly frozen in liquid nitrogen and ground to a fine powder in liquid nitrogen using an IKA A11 basic analytical mill. RNA was extracted in three technical repeats using the Norgen Biotek RNA Extraction Kit according to the manufacturer’s instructions. Samples of RNA (total of 12; three T0 white, three T0 green, three IBA white, three IBA green) were sent on dry ice to Macrogen, South Korea, for deep sequencing. The raw reads (100 bp) were subjected to a filtering and cleaning procedure. The FASTX Toolkit (http://hannonlab.cshl.edu/fastx_toolkit/index.html, version 0.0.13.2) was used to trim read-endnucleotides with quality scores <30, using the FASTQ Quality Trimmer, and to remove reads with less than 70% base pairs with a quality score ≤30 using the FASTQ Quality Filter. Paired-end reads were aligned to the *Persea americana* var. *drymifolia* (Mexican avocado) genome from the National Center for Biotechnology Information (NCBI) (ASM803378v1; https://www.ncbi.nlm.nih.gov/assembly/GCA_008033785.1/) using Tophat2 software (v2.1) [83]. Gene abundance was estimated using Cufflinks workflow (v2.2), including Cufflinks, Cuffmerge and Cuffquant [84]. Differential expression analysis was performed with the DESeq2 R package [85] in the R environment. PCA clustering was done based on normalized counts using the tool: https://pcago.bioinf.uni-jena.de/. Genes that were more than 2-fold differentially expressed with a false discovery rate (FDR)-corrected statistical significance of no more than 0.05 [86] were considered differentially expressed. The transcriptome sequences based on the genomes were used as a query list for a homology search in the NCBI non-redundant (nr) protein database that was carried out with the DIAMOND program [87]. The search results were imported into Blast2GO version 4.0 for gene ontology (GO) assignments [88]. Homologous sequences were also identified by searching the Swiss-Prot and *Arabidopsis* databases (TAIR, http://www.arabidopsis.org) with the BLASTx tool [89] and an *E*-value threshold of 10^−5^. Hierarchical cluster analysis of heatmaps was performed with the ClustVis tool [90]. Unit variance scaling was applied to rows. Pearson correlation was used for the distance calculations, and average linkage criterion was used to cluster the genes. The sequence data was loaded to the NCBI SRA data base ID: PRJNA660369.

### 4.6. Compression Analysis

Compression tests were carried out using a TA.XT plusC instrument (Stable Microsystems, UK) with a flat probe of 5 mm diameter. The maximal strain required to compress the stems by 15% of their diameter was calculated and expressed in Newtons (N).

### 4.7. Chlorophyll Measurements

Chlorophyll measurements were done using a portable MC-100 instrument (Apogee Instruments, Inc). Chlorophyll concentrations (µmol of chlorophyll per m^2^) were measured from intact leaf samples in 5 plants of each white or green each time branches were collected.

### 4.8. Auxin Measurements

To measure auxins and auxin conjugates, at least 10 different branches from each treatment were pooled and frozen in liquid nitrogen immediately after excision, ground, freeze-dried and sent (3 technical repeats of 100 mg each) to the Plant Biotechnology Institute, National Research Council, Saskatoon, Canada, for hormone profiling. Briefly; deuterated forms of the hormones and conjugates were used as internal standards. Analysis was performed on a UPLC/ESI-MS/MS utilizing a Waters ACQUITY UPLC system, equipped with a binary solvent delivery manager and a sample manager coupled to a Waters Micromass Quattro Premier XE quadrupole tandem mass spectrometer via a Z-spray interface. MassLynx™ and QuanLynx™ (Micromass, Manchester, UK) were used for data acquisition and data analysis. The analyses utilized the Multiple Reaction Monitoring (MRM) function of the MassLynx v4.1 (Waters Inc) control software. The resulting chromatographic traces were quantified off-line by the QuanLynx v4.1 software (Waters Inc) wherein each trace is integrated and the resulting ratio of signals (non-deuterated/internal standard) is compared with a previously constructed calibration curve to yield the amount of analyte present (ng per sample). Calibration curves were generated from the MRM signals obtained from standard solutions based on the ratio of the chromatographic peak area for each analyte to that of the corresponding internal standard.

### 4.9. Sugar Analyses

The soluble sugars sucrose, glucose and fructose, and starch were analyzed as previously described [91,92]. Briefly, samples of white or green branches included three biological repeats of 3 gr. Each repeat was composed of three different branches, 1 gr each, total of 9 gr of each branch types. These were stored separately in 5 mL 80% ethanol in 6 separate tubes. Soluble sugars were extracted in three sequential incubations at 70 °C. The ethanol was pooled for each sample and was then evaporated overnight at 55 °C. Remaining sugars were dissolved in 1 mL H_2_O and filtered through a 0.25-μm micropore filter. Sucrose, glucose and fructose levels were resolved by high-pressure liquid chromatography (HPLC). The HPLC system consisted of a Shimadzu LC10AT solvent-delivery system and a Shimadzu RID10A refractive index detector. Separation was carried out on an Alltech 700 CH Carbohydrate Column (Alltech, USA) maintained at 90 °C with a flow rate of 0.5 mL/min, according to the manufacturer’s recommendations. For starch analysis, the plant tissue was dried overnight at 60 °C and then soaked in 6 mL H_2_O and heated to 121 °C in a pressure autoclave for 1 h for starch extraction. Extracted starch was digested by overnight incubation with 10 mg/mL β-amyloglucosidase (Sigma) at 55 °C. The levels of resulting glucose were measured by Sumner assay.

## 5. Conclusions

Based on the high level of expression of numerous ethylene responsive transcripts, it is concluded that high ethylene activity existed in the etiolated branches. This is at least partially the cause of cell-wall modifications, which together led to softer cell walls in the etiolated branches. After 1 to 2 weeks in the light, some differences occur, but the green branches do not yet resemble green branches that have never grown in the absence of light; some of the etiolation-derived traits are still there, at least to a certain extent. Their cell walls are not yet more lignified than those of the white branches, and they are probably not yet too rigid. These likely make the green branches more prone to adventitious root formation than the white ones. It should be noted that, beyond rooting compatibility, the changes occurring during de-etiolation might also make the green branches more resistant to the long incubation on the rooting table and reduce their sensitivity to pathogens, which might contribute to their higher survival and enable better rooting rates.

## Supplementary Materials

The following are available online at https://www.mdpi.com/2223-7747/9/11/1481/s1, Figure S1: Zoom-in on the cambium tissues of white and green cuttings, Figure S2: A. Number of reads and percent mapping to the Hass avocado genome. B. The clustering of the results of the different samples. Figure S3: Heatmap of differentially expressed transcripts related to auxin.
