# Supplementary figures and images for: Short De-Etiolation Increases the Rooting of VC801 Avocado Rootstock
Source: Plants (Basel). 2020 Nov 3;9(11):1481. doi: 10.3390/plants9111481 (PMC7693756; doi:10.3390/plants9111481)

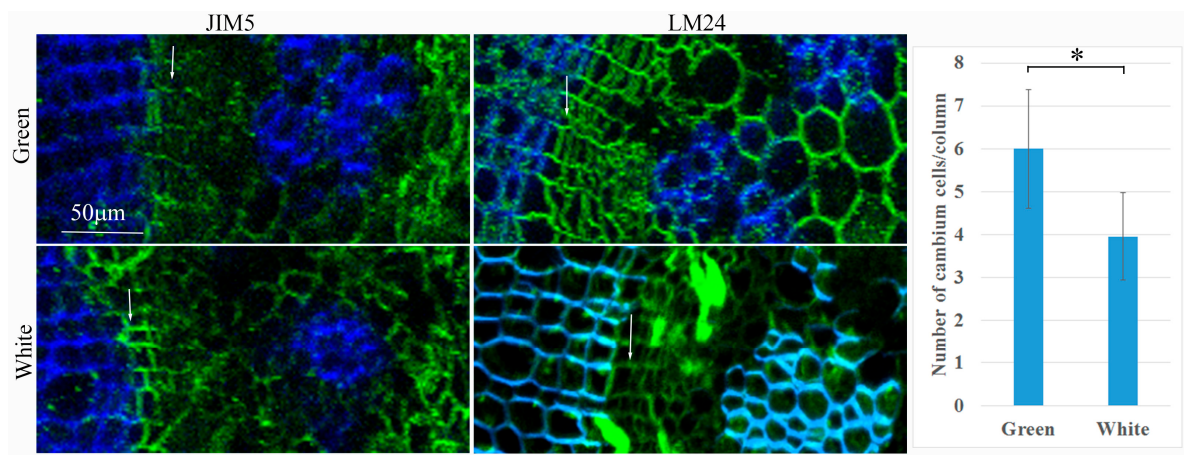

Figure S1.

Supplement: Supplementary file 1 [file plants-09-01481-s001.pdf]
